# Near Infrared Imaging As a Method of Studying Tsetse Fly (Diptera: Glossinidae) Pupal Development

**DOI:** 10.1093/jisesa/iew047

**Published:** 2016-07-07

**Authors:** Zelda R. Moran, Andrew G. Parker

**Affiliations:** ^1^ Insect Pest Control Laboratory, Joint FAO/IAEA Programme of Nuclear Techniques in Food and Agriculture, International Atomic Energy Agency, P.O. Box 100, 1400 Vienna, Austria (zelda.moran@gmail.com; a.g.parker@iaea.org)

**Keywords:** Glossina palpalis gambiensis, time-lapse video, pupation, pupariation, sex separation

## Abstract

Near infrared (NIR) photography and video was investigated as a method for observing and recording intrapuparial development in the tsetse fly *Glossina palpalis gambiensis* and other Muscomorpha (Cyclorrhapha) Diptera. We showed that NIR light passes through the puparium, permitting images of the true pupae and pharate adult to be captured. Various wavelengths of NIR light from 880 to 1060 nm were compared to study the development of tsetse fly pupae from larviposition to emergence, using time-lapse videos and photographs. This study was carried out to advance our understanding of tsetse pupal development, specifically with the goal of improving a sorting technique which could separate male from female tsetse flies several days before emergence. Separation of the sexes at this stage is highly desirable for operational tsetse sterile insect technique control programmes, as it would permit the easy retention of females for the colony while allowing the males to be handled, irradiated and shipped in the pupal stage when they are less sensitive to vibration. In addition, it presents a new methodology for studying the pupal stage of many coarctate insects for many applications. NIR imaging permits observation of living pupae, allowing the entire development process to be observed without disruption.

Tsetse flies (Diptera: Glossinidae) are the sole vectors of African Trypanosomosis, a devastating parasitic disease which threatens both humans and livestock in sub Saharan Africa. Though a great deal of research on ecology, physiology, genetics, and reproductive biology of tsetse flies has been carried out over the past 100 yr, little is known about the pupal stage of the insect. Knowledge of the pupal development process could be critical for a variety of tsetse control methods, as vector control remains the most important method of nagana and sleeping sickness prevention (Schofield and Kabayo 2008; WHO 2013).

One of the most effective methods of tsetse population eradication is the Sterile Insect Technique (SIT), which involves mass rearing and release of sterilized male tsetse flies into isolated populations of wild flies. The sterilized males inseminate wild females, which typically mate only once in their lifetime, meaning that insemination by an infertile male eliminates the chance of new progeny. As sterilized male release continues, the ratio of sterile males to wild males increases, and as fewer females are able to produce offspring, the population declines to zero (Vreysen 2001; Feldmann et al. 2005). SIT depends on large numbers of healthy males being available for sterilization, and may involve shipment of pupae across long distances to the release site. Due to the slow rate of reproduction of tsetse, it is necessary to retain the females for colony maintenance and only ship males for release. At present, males and females can only be separated upon adult emergence; this is facilitated to some extent by the relative protogyny but there is still considerable overlap in the emergence. Relying on this results in the males being within a few hours of emergence (pharate adult) when they are most vulnerable to vibration damage, and to prevent emergence and permit handling and irradiation the pupae must be chilled. Extended chilling of pharate adult males during long distance transport reduces quality and increases mortality (Pagabeleguem et al. 2015). Any system that enabled the sexes to be separated earlier in development would greatly facilitate handling and improve the released male fly quality. Improved knowledge of the pupal development will help with the development of such a system.

Changes in pupal eye color have already been identified (Rabossi et al. 1992; Resilva and Pereira 2014) as a useful metric for timing irradiation in male fruit flies. Rabossi and Quesada-Allue (1993) also identified UV light as a tool for visualizing eye pigmentation, which fluoresces under UV and can be used to accurately age pupae and during the transition from larva to pharate adult. Thus, understanding pupal development to improve SIT is not a new idea, but has not been studied in tsetse flies. Knowledge of the pupation process in medfly, tsetse, and other ecologically important insects may be useful to a variety of applications for SIT and beyond.

The vast majority of work on tsetse pupal development was completed decades ago, and to our knowledge, no work examining development of structures inside the pupa has been carried out on living samples. There is a deficit of data not only for tsetse pupae, but also in the pupal stages of many medically important insects, suggesting a potentially important weakness in the field of public health entomology. The aim of this study was to: (1) improve understanding of the pupal stage of tsetse flies, in the context of sex separation and (2) present a new methodology for studying pupal stages of insects which undergo coarctate metamorphosis.

Bursell (1958, 1959) published the most complete work on tsetse pupal development. He describes three stages in the pupal period:

*Stage I*. *The third instar puparium.* This stage lasts for the first 24 h after larvipostion. The larva remains motile for ∼20 min after larviposition, allowing it to burrow into the soil. The larva then becomes smooth and rounded as the cuticle hardens, forming what will become the puparium and darkens as it sclerotizes. Figure 1 shows a larva from the *Glossina palpalis gambiensis* Vanderplank (Diptera: Glossinidae) colony in Seibersdorf, Austria, which sclerotizes and turns black over the course of 24 h. The external appearance then remains the same until the adult fly emerges.

**Fig. 1.**
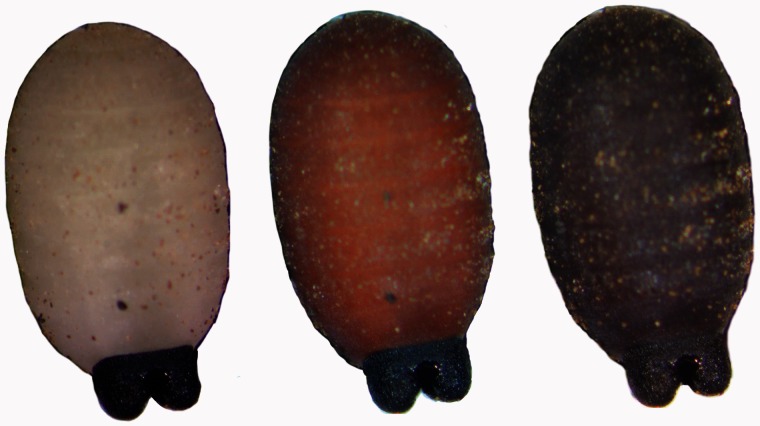
Transformation of a third instar *Glossina palpalis gambiensis* larva into a puparium over the first 24 h after larviposition.

*Stage II*. *The fourth instar larva stage*. This lasts from days 2 to 4, when the larva retracts from the inside of the puparium.

*Stage III*. *Pupa and pharate adult*. The remainder of the pupal period (days 5–30), constitute the true pupa and pharate adult stage. The fourth instar larva molts within the puparium around day 5, leaving an exuvium on the inner walls. Thus, although the “pupal period” is considered to be the time between the hardening of the larval cuticle until the emergence of the adult fly, it is not until day 5 that the true pupa is formed. According to Bursell (1958), “Between the hypodermis and the pupal skin there is secreted a quantity of clear fluid below which the cuticle of the adult is formed. This fluid is gradually reabsorbed in the course of further development, until at a day or two before emergence no liquid remains and the pupal skin dries out.” The last two days of the pupal period are referred to as “pre-emergence”, and the fly is termed a pharate adult.

There have been several studies investigating length of tsetse pupation in relation to temperature or other environmental factors. Jackson (1949) and Phelps and Burrows (1969a,b) [discussed by Hargrove (2003) and Childs (2011)], e.g., describe a linear relationship between temperature during pupation and length of the pupal period—pupae kept in warmer environments have significantly shorter pupal periods. Hargrove (1990) discusses the impact of pupal dehydration as a driver of early-stage pupal mortality and pupal water balance is modeled as a function of time by Childs (2009, 2012). A method of viewing structures of the growing fly without interrupting development can give further insight into what effects these environmental conditions have, and at what stage of pupation.

Near infrared (NIR) spectroscopy has been used to examine various samples to determine specific properties, including the presence of insects in seeds and the composition of grain (Dowell et al. 1998; Baker et al. 1999; Baye et al. 2006). This has been developed into systems for the identification of the stage of development and even specific identity of the insects (Dowell et al. 1999, 2010; Perez-Mendoza et al. 2002, 2004; Aldrich et al. 2007; Mayagaya et al. 2009; Reeves et al. 2010; Sikulu et al. 2010; Aw et al. 2012) and for the identification of the sex in tsetse fly pupae some days before emergence (Dowell et al. 2005). This work on detecting insects in seeds and sexing tsetse pupae used entirely empirical analysis of the spectra to develop a calibration. Having no specific theoretical basis for the empirical relationship, it was speculated that the spectrometer was detecting various specific chemical species in the samples related to physiological development in the flies (Dowell et al. 2005) but this was never confirmed.

Initially, standard laboratory spectrophotometers were used for studies involving NIR light in entomology, but subsequently a number of specific instruments have been developed, of varying sophistication (Dowell et al. 2006). Recent systems utilize the characteristic emission spectra of selected light emitting diodes (LEDs) with a standard silicon or indium–gallium–arsenide (InGaAs) photodiode to provide a restricted range of spectral information rather than using a much more expensive, full spectrum spectrometer (Haff et al. 2013; Pearson et al. 2013). One such system (LED Vis-NIR Seed Sorter System, National Manufacturing, Lincoln, NE) was obtained for testing in our laboratory as a pupal sex sorter. The device appeared to have promise, but we observed it to be unreliable and it was not understood on what characters the sorting was based.

InGaAs detectors have a broad response in the infrared (Hamamatsu Photonics K.K. 2015), but their sensitivity is lower than that of silicon and image capture arrays are expensive with low resolution. Silicon sensors, by contrast, have a near linearly increasing response with wavelength from ∼360 nm to a peak ∼1000 nm (Darmont 2009) then falling rapidly to zero by 1100 nm, and the megapixel arrays are cheap and readily available. In order to avoid color biases, an infrared filter is incorporated into all standard cameras, but by removing this filter the image sensor becomes usable in the NIR to ∼1080 nm. LEDs are available with a wide range of output wavelength, including many compact, high power diodes that emit in the NIR. LEDs in the 800–950 nm range are typically used in infrared remote control applications and are readily available. Longer wavelength LEDs are less widely available, but LEDs with a wavelength ∼1050 nm are still only a few Euro each.

It was hypothesized that by using a standard silicon image chip with the NIR filter removed and a range of different NIR LEDs it would be possible to determine what was being detected by the sorting machine, thus providing a better understanding of the causes of variation in the sorting efficiency.

## Materials and Methods

### Pupae

Tsetse pupae were taken from the *Glossina palpalis gambiensis* colony at the Insect Pest Control Laboratory (IPCL), Joint FAO/IAEA Division of Nuclear Techniques in Food and Agriculture, Seibersdorf, Austria and were stored in an environmental chamber (ES2000, Environmental Specialties, Zellweger Luwa Group, 4412 Tryon Road, Raleigh, NC) or temperature controlled holding room at 24 ± 0.5 °C, 75 ± 5% relative humidity, throughout the study. The colony was first colonized at Maisons-Alfort, France in 1972 using pupae collected in Guinguette, Burkina Faso, and transferred to the Centre International de Recherche-Développement sur l’Elevage en zone Subhumide (CIRDES), Bobo Dioulasso, Burkina Faso in 1975. The strain has been reared at the IPCL, Seibersdorf, Austria since 2009 from pupae derived from the colony maintained at CIRDES (Mutika et al. 2013). All flies were fed using a silicon membrane feeding system (Feldmann 1994). When maintained at 24 °C, the pupal period for *G. p. gambiensis* is quite constant, with females emerging between days 32 and 34, and males emerging from days 34 to 37.

Pupae of *Musca domestica* L. (Diptera: Muscidae) were obtained from a colony at the Institute of Zoology, Slovak Academy of Sciences, Bratislava, Slovakia and of *Bactrocera dorsalis* (Hendel) (Diptera: Tephritidae) from a colony held in the IPCL.

### Photography and Video

**Fig. 2.**
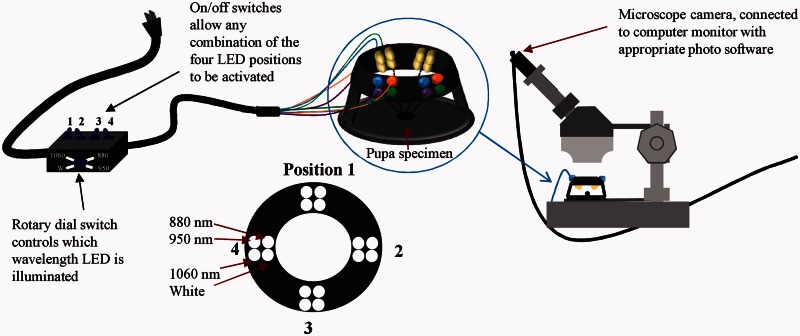
Schematic diagram of the experimental setup.

The equipment set up for NIR imaging is shown in Fig. 2. Pupae were photographed under a dissection microscope (Stemi SR, Zeiss, Carl-Zeiss-Strasse 22, 73447 Oberkochen, Germany) using a USB eyepiece camera (Dino-Eye eyepiece camera, AM423X, AnMo Electronics Corp., Taiwan) with the infrared filter removed. The pupae were illuminated under LEDs of 880 nm (SFH 485-2, Osram Opto Semiconductors, Regensburg, Germany), 950 nm (TSAL6100, Vishay, Malvern, PA), 1060 nm (ELD-1060-525, EPIGAP Optronic GmbH, Berlin, Germany), and white (LTW-2S3D8, Lite-On Inc., Milpitas, CA) that were mounted on a circular stand in a ring around the pupa. The stand was made to fit inside the stage of the dissection microscope. The lights fit into holes inside a ring 30 mm above the base. The LEDs were arranged with one 880, 950, 1060 nm, and white LED placed in each of four equally spaced banks around the center of the stage. A rotary switch controlled which wavelength LEDs would illuminate: white, 880, 950 or 1060 nm infrared. An on/off switch was also installed for each of the four positions around the stand, so that any combination of the four could be illuminated. The LEDs were driven at 95 mA for the three NIR LEDs and 30 mA for the white LEDs using LM317LZ constant voltage sources as current drivers from a 7.5-V supply.

Rigid strips of cardboard were glued to the bottom of the stand, so that standard 75 mm×25 mm glass microscope slides could slide in and out of the stand, allowing the subject to be repeatedly placed in the same position under the camera.

### Photographs

**Fig. 3.**
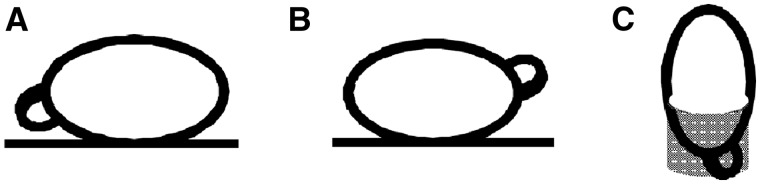
Orientation of the pupae for observation under a stereo microscope: (A) ventral view (ventral side facing up), (B) dorsal view (dorsal side facing up), and (C) anterior view (head facing up).

Some pupae were secured onto glass slides using cyanoacrylate glue, whereas others were kept loose so that they could easily be rotated under the microscope. In this way, some pupae could be photographed repeatedly in exactly the same position, ensuring a smoother series of photographs, whereas full views could be obtained of others. For those secured on slides, three positions were used:

Ventral view (Fig. 3A): The pupae were placed with the polypneustic lobes pointing down. Viewed from above, this position showed the ventral surface with wings, proboscis, legs, and eyes all visible.

Dorsal view (Fig. 3B): The pupae were placed with the polypneustic lobes pointing up, giving a view of the dorsal surface. Wings, eyes, frons and hairs and body markings were visible in this position.

Anterior view (Fig. 3C): The pupae were secured in small rings of plastic tubing, which were glued vertically onto the slide, giving an anterior, head-on view of the pupae from above. An air hole was cut at the bottom of the tube for the polypneustic lobes. Eyes, the base of the antennae, and the frons were visible.

In total, 50 pupae were photographed for the first stage of the study. Thirty-three pupae were mounted on slides using cyanoacrylate glue and were photographed under each wavelength of NIR—880, 950, 1060, and white every week-day from larviposition until emergence. About 10 pupae were fixed in ventral view, 9 in dorsal view, and 14 in anterior view.

This allowed pictures to be taken with the pupa in the same position every day. Pupae were placed on slides in two sets, and spaced in age by several days, ensuring that there were no gaps in the set of ages documented. Seventeen pupae were not mounted on slides, but were photographed in all three positions each day. Once a complete set of photographs had been taken for each pupa, photos were viewed at their original resolution on screen using DinoLite image viewer software and all visible developments were noted. Stages of development were observed and compared between sets, and a basic time line of development was identified. In the second stage of the study, ∼100 additional pupae were photographed in ventral position on days 22–29.

### Video

Time-lapse video was taken using a digital USB camera (Dino-Lite Pro AM413-FITA, AnMo Electronics Corp., Taiwan), equipped with of 850 nm LEDs and no NIR filter. The camera was fixed over four pupae, all in ventral view, since the timing of wing pigmentation was noted as the most obvious difference in development between the sexes. The video was set when the pupae were ∼2 h old, and remained running until all adult flies had emerged. The time interval was set at 5 min 36 s, on photo mode rather than video mode, so still photos were also produced. The resultant video was processed using Adobe After Effects to insert captions and to reduce time-lapse flicker by equalizing color over time.

## Results

**Fig. 4.**
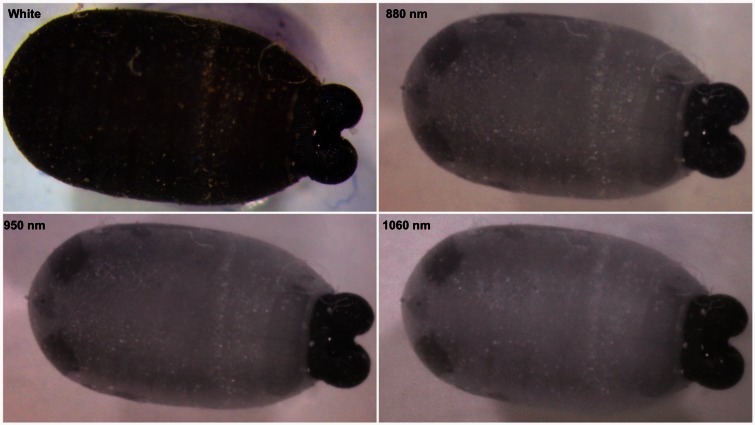
Comparison of a 28-d-old puparium of *Glossina palpalis gambiensis* under various light sources, white LED, 880 nm LED, 950 nm LED, and 1060 nm LED. The same structures are visible under all of the NIR wavelengths.

One puparium, illuminated with each of the NIR LEDs (880, 950 and 1060** **nm) is shown in Fig. 4. The 1060 nm photos were slightly clearer and showed a few more details than the other wavelengths. All subsequent images and results are based only on the 1060 nm illumination except where noted.

### Basic Timeline of Pupal Development

Figure 1 shows a larva from the *G. p. gambiensis* colony in Seibersdorf, Austria, which sclerotizes and turns black over the course of 24 h.

Structures and appendages begin to appear under all wavelengths of NIR tested (880, 950 and 1060 nm) on day 6. The visible developmental events are as follows;

*Day 0*: Small movements still visible through puparium in video. No structures visible

*Day 6*: Appearance of legs and eyes, continuing to darken and sharpen until day 10, remaining almost the same until day 21.

#### Appearance of frons

*Day 20*: Darkening of eyes (days 20–27)

*Day 24*: Darkening of wings (days 24–27)

*Day 25*: Darkening and sharpening of legs and proboscis (days 25–35. Legs fully darkened by day 28)

**Fig. 5.**
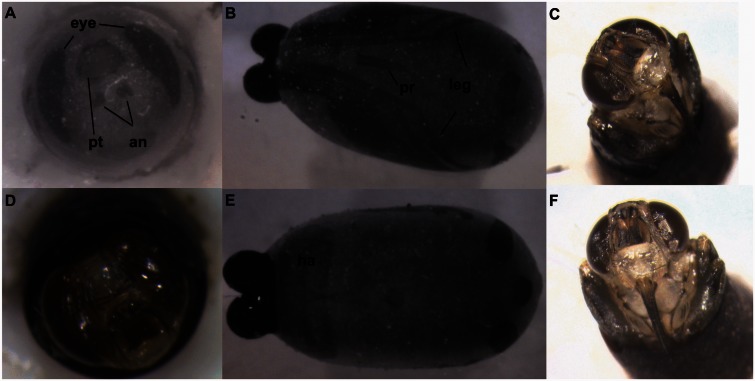
Images of fully developed, 30-d-old pupae under 1060 nm NIR light and corresponding photos of the dissected pharate adult. In image (A), the eyes are visible and are fully pigmented at this stage. The ptilinum and antennae are also very prominent. Image (B) is a ventral view, and shows wings on the sides of the pupae, the proboscis in the center, and the legs spreading to each side. The ptilinum is also visible, as is a small section of the eyes. Images (C), (D), and (F) show the same pupae with the top of the pupa removed so that the structures are visible. Image (C) and (F) show the legs and proboscis visible in image (B) under NIR, and image (D) shows the eyes, ptilinum, and antennae visible under NIR in image (A). Image (E) shows the dorsal view, with the eyes, ptilinum, and hairs visible on the body. an, antennae; pt, ptilinum; pr, proboscis; ha, hair.

Images of fully developed pupae are shown under 1060 nm NIR illumination in Fig. 5.

**Fig. 6.**
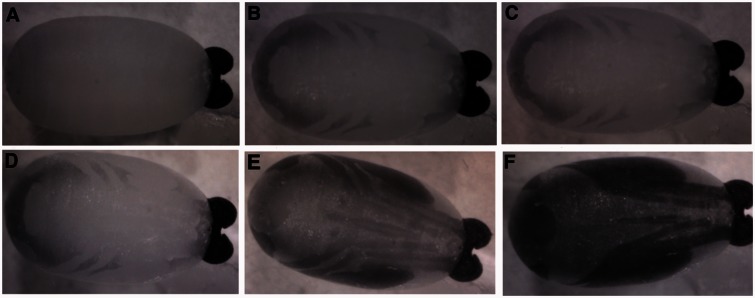
Ventral view of developing *Glossina palpalis gambiensis* puparia under 1060 nm NIR light: (A) Day zero, no structures visible; (B) Day 6, legs visible; (C) Day 12, no further developments; (D) Day 20 appendages slightly sharper; (E) Day 27, pigmentation has occurred in legs, wings, and eyes; and (F) Day 32, legs, wings, antennae, frons and eyes fully pigmented.

**Fig. 7.**
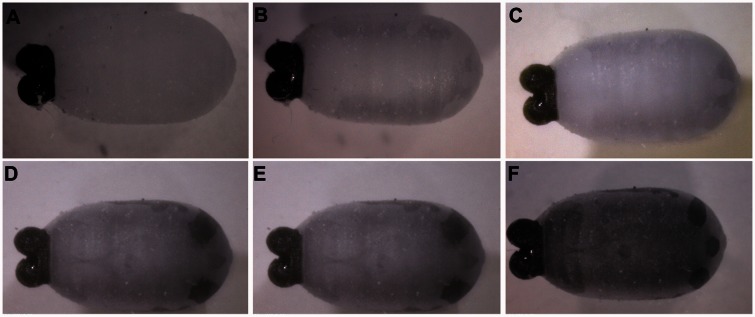
Dorsal view of *Glossina palpalis gambiensis* puparia under 1060 nm NIR light: (A) Day zero, no structures visible; (B) Day 6, eyes visible, frons visible; (C) Day 11, No further developments; (D) Day 19, No further developments; and (E) Day 28, pigmentation in wings, eyes, frons, and bristles is visible. We also observe an unidentified branching pattern on the back; (F) Day 33, bristles are darkened and more visible near the posterior end. Eyes appear more defined and smaller because of air pockets between them and the puparium, which seem to develop just before emergence.

**Fig. 8.**
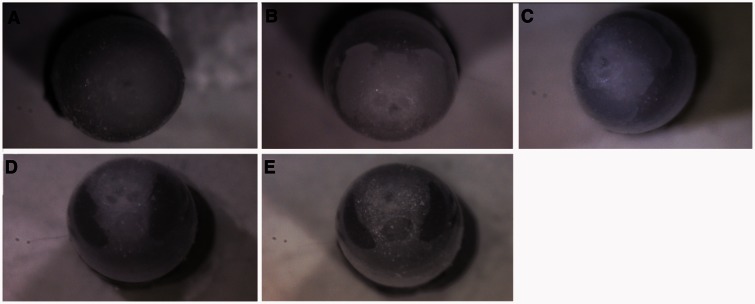
Anterior view of *Glossina palpalis gambiensis* puparia under 1060 nm NIR light: (A) Day zero, no structures visible; (B) Day 6, eyes visible, frons visible, no pigmentation; (C) Day 20, eyes begin to darken; (D) Day 25, eyes darkened; and (E) Day 33, eyes and frons darkened, air bubbles begin pushing tissue away from the inside of the puparium before emergence.

**Fig. 9.**
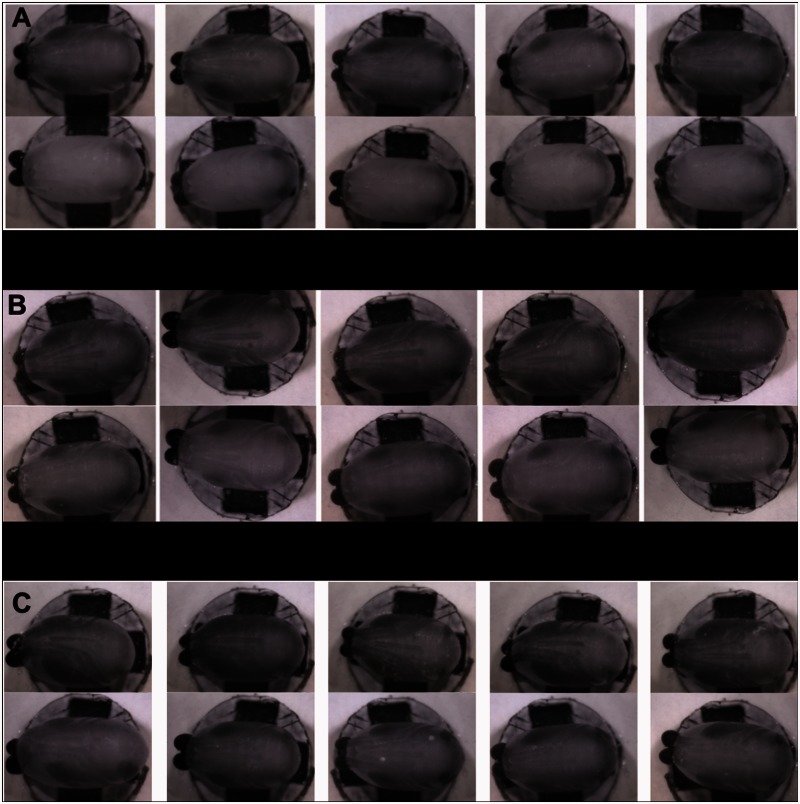
(A) Pupae shown at day 25 of development. Females are in the top row, males are in the bottom row. Females show some pigmentation in the wings and legs, whereas males do not. (B) Pupae shown at day 26. Females on the top row. Females show dark pigmentation in both wings and legs, whereas male wings are only beginning to darken. Legs are not yet pigmented in males. (C) Wings and legs are very dark in females, and darker in males compared to day 26. The difference between male and female is becoming less obvious.

**Fig. 10.**
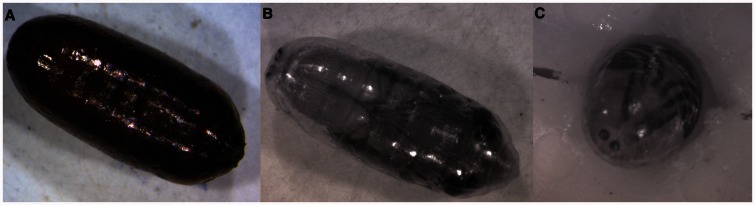
*Musca domestica* puparium under white light (left) and 1060 nm IR light (center, right).

**Fig. 11.**
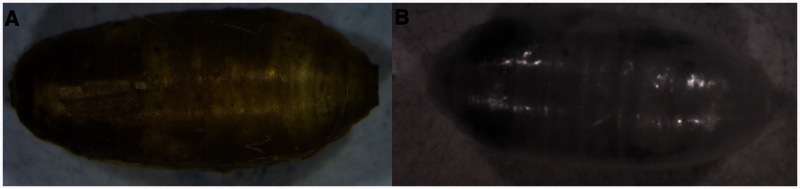
*Bactrocera dorsalis* puparium under white light (left) and 1060 nm NIR light (right).

Figures 6–8 show sequences of photographs showing the development of pupae in ventral, dorsal, and anterior positions. Figure 9 shows the difference between male and female tsetse pupae at day 26. Figures 10 and 11 show photographs taken of *M. domestica* and *B. dorsalis*, respectively, to illustrate this technique’s applicability to other species.

It was quickly observed that the ventral view, under 1060 nm, revealed the most structures, including a notable difference in timing of wing pigmentation between males and females.

### Tsetse Pupal Development

#### Ventral view (Fig. 6)

If observed during the first 24 h, the third instar puparium does not reveal any structures or appendages, but slight heartbeat-like movement is visible under video or if observed live under the microscope. On day 6, the outline of eyes, legs, and frons appear, in perfect keeping with the findings of Bursell (1958) that the pupa retracts from the inside of the puparial shell on this day. No further changes are observed until pigmentation begins around day 24 for females and 27 for males. Pigmentation begins in the wings and spreads through the legs and eyes. By day 28, eyes, wings, and legs are pigmented and will continue to darken until pre-emergence (day 32 onward).

#### Dorsal view (Fig. 7)

Similar to ventral position, the first outlines of structures are visible on day 6, when the eyes, frons, and a part of the abdomen become visible. Again, no further changes occur until pigmentation begins around days 24–27. Eyes, frons, and wings darken, and bristles become visible on the abdomen. In the days before emergence, all structures darken in color, but eyes and frons may shrink in size and sometimes disappear altogether, presumably due to air pockets developing and separating the pharate adult from the inside of the pupal shell in preparation for emergence. This would also be in keeping with the findings of Bursell (1958, 1959) that moisture is absorbed during the days leading up to emergence. An unidentified tree-shaped pattern of markings was often visible on the dorsum of the pupa (Fig. 7).

#### Anterior view (Fig. 8)

This view was perhaps the least revealing, simply because only eyes and frons were visible. Again, outlines are clear on day 6, and pigmentation begins slightly around day 21 or 22, is clear by day 23, and darkens until day 28. Again, the apparent size of the eyes often shrinks in the days leading up to emergence. Antennae are often clearly visible from this angle as well.

The pupae in these photographs were not fixed to slides with cyanoacrylate glue, but were unattached and simply positioned each day to be photographed. This is because 10 out of the 15 pupae which were fixed in this position died during the early stages of development. Lack of oxygen was not the cause of this, as a space had been provided in the small piece of tubing that was used to support the pupae.

All visible events were recorded in a time-lapse video of the ventral side of two male and two female tsetse flies (Supp Video S1 [online only]).

### Distinguishing Males and Females

It is well established that female tsetse emerge 2–3 d before males and that the frons is narrower in males. It was thought, therefore, that female eyes would darken before males, and that the pupa could be sexed by measuring the distance between the eyes. If observed closely, it did appear that this could be possible, but we were unable to achieve reliable results using this method. However, a difference in wing pigmentation was obvious. On day 25, females all showed some pigmentation in wings, and the beginning of pigmentation in legs and proboscis, whereas males still appeared white. On day 26, wings on many males had started to darken, but females showed far more pigmentation, both in wings and legs. On day 27, the difference became less obvious, with females still appearing to have significantly darker wings, but at less of a contrast to males.

To further explore this difference, sets of 20 pupae from days 22 to 29 were photographed under NIR light, then dissected and sexed. The results showed a consistent difference: female wings darken before males. This allowed 26-d-old pupae to be identified as male or female. Figure 9 shows sets of female tsetse pupae at days 25–27 compared to male pupae of the same age. The difference is striking, and contradicts the assumption of Bursell (1959) that if there were a difference in development between males and females it would only be by a third of a day.

### Applications Beyond Tsetse

NIR imaging is not limited to tsetse research. *M. domestica* (Fig. 10) and *B. dorsalis* (Fig. 11) puparia were both transparent under NIR light, showing similar features to those seen in tsetse flies. A 24-h time lapse video of *M. domestica* (Supp Video S2 [online only]) shows dramatic darkening of wings, legs, and eyes. It seems likely that pupal stages of many members of the muscomorphan (cyclorrhaphan) Diptera and other insect groups could be studied using NIR imaging. This technique could be applied to any insect which undergoes coarctate metamorphosis. In addition NIR has long been used to study grain properties such as moisture and protein content, and infestation by insects (Pearson et al. 2001; Yao et al. 2010; Sundaram et al. 2012; Lee et al. 2014), but this has been limited to spectral analysis and to our knowledge NIR images have not been used in the field of entomology

## Discussion

This study provided new insight into the timeline of tsetse pupal development and showed a clear difference in the timing of wing pigmentation in male and female tsetse flies. The difference in timing of the wing pigmentation coincides with the time when the various NIR sorting systems are best able to distinguish female from male tsetse and for the first time indicates what character the sorting systems are likely to be detecting. We anticipate that it will be possible to develop sexing systems based on NIR imaging of wing pigmentation.

The silicon sensors in the two cameras limit the range of NIR that can be used to a maximum wavelength of ∼1100 nm. The three NIR LEDs (880, 950 and 1060 nm) were selected to approximately cover the range from the end of the visible spectrum (∼700 nm) to near this limit., but in practice little difference was observable at the wavelengths used in this study and it seems that any available NIR light source in this range could be used.

Interestingly, structures were visible under NIR long before they would be visible in a dissected sample. For example, pupae dissected at 10 d of age had no discernable structures and consisted only of loose, white tissue. NIR photographs taken at this age, however, clearly show legs and the outline of eyes. As previous work on pupal development has consisted only of either external observation or dissected samples, this technique allows much more to be observed of the stage of pupal development.

So far, the NIR sorting systems have proved very variable in their performance, hampering their development. Dowell et al. (2005) showed high sorting efficiency, but this was only achieved sporadically with the system sorting correctly 1 d but failing the next with pupae of the same age and with the same calibration. It is clear from the present work that the orientation of the puparium during reading is important as the melanizing wings are only clearly visible from the ventral side. We plan now to develop calibrations based on specific orientations and to investigate mechanisms for ensuring the consistent presentation of the puparia to the reading system to ensure the wings are visible to the system.

It is our hope that this technique will be adapted for many purposes in the field of entomology and beyond. The equipment and methodology used are simple and reproducible in any laboratory, though it should be noted that as temperature greatly impacts the length of the pupal period (Leak 1998), the timing of development found in this study cannot be directly applied to any other colony or species.

## SUPPLEMENTARY DATA

Supplementary data are available at *Journal of Insect Science* online.

## Supplementary Material

Supplementary Video S1

Supplementary Video S2
